# Trismus Neonatorum

**Published:** 1880-04-20

**Authors:** 


### *TRISMUS NEONATORUM—RECOVERY.*

On January 20th, after a tedious labor of 24 hours, Mrs. M——, aged 20 years, white, was delivered of her first-born mature female child.

At birth the funis was twice around the neck, which accounts for the delay in delivery; was in a state of asphyxia and exceedingly cyanotic. It took half an hour to resuscitate the infant. During that process a large quantity of frothy mucus, slightly tinged with blood, poured from the nostrils.

Eight hours after birth unilateral convulsions were observed of the left side. This was at 8 P.M.; twelve hours after it was general, with frequent spasms; rigid lower jaw, with mouth sufficiently open to admit a finger; difficult breathing, livid countenance, clenched hands, with thumbs flexed into the palms, and produced on the slightest motion, commencing with a little scream.

When seen, was immediately recognized as an old enemy that had not been witnessed by me for twenty-four years previous, who had vanquished me every time, and I had hoped never to meet again. Have treated several cases among the negroes in Louisiana, but no means then used prevented a fatal termination. It has been stated that these cases "invariably occurred on the sea-coast, from cold and damp weather, and unknown in the interior of the country."

All the cases previous to this one were seen in warm weather, in the south, not less than two hundred miles from the sea-coast.

The surroundings in this case were all that could be desired—cold and dry, with thermometer about 32° Fahrenheit. I did not fail to warn all, except the mother, that they must prepare for a fatal result.

*Treatment.*—Thinking that it might relieve the brain, free catharsis was induced, with calomel and castor oil; after which five-gain doses of bromide of potassium in sweetened water; very soon observing no improvement, had recourse to the following recipe:

℞ Physostigma.......................................... gr. ss.

Glycerine.......................................... ʒ ij.

Aqua.................................................f ʒ vi.

Dose, thirty drops every four hours.

After three doses the convulsions were less severe, and after six had been given the paroxysms came on with much longer intervals, milder and of shorter duration; so that by the time eight doses had been given, they had entirely ceased and did not return, although the above was continued in half doses every six hours for the next twenty-four hours.

During this treatment the child was nourished with milk and barley water; although the feeding would induce convulsions, yet the child swallowed without difficulty. After the attack was overcome, for four or five days the child had not the power to nurse, so that Knapp's breast pump was used and fed to the child until she was able to help herself.

It was, a week after birth, as well and healthy as any child of that age.

It may be as well to state that particular directions were given during the attack that the child should be laid on its side and not on the back, in order to avoid pressure on the occiput.

Feeling that I had a fearful case to deal with, was compelled without delay to make use of desperate means (many would consider the dose too large for a new-born infant) before the little patient was exhausted or became comatose.

Had the poisonous effect of the drug exhibited itself by tremulousness and loss of power of the extremities, becoming limp and flaccid, indicating the approach of general paralysis, should have used chloral as an antidote.

This is the first case of the kind in which I have seen the calabar bean used, and hoping it may prove as useful to others as it was in this, has induced me to report it.—*Proceedings of the Medical Society of the County of Kings.*

